# AUR1 and its pals: orchestration of intracellular rhizobia infection in legume for nitrogen fixation

**DOI:** 10.1007/s00299-023-02979-x

**Published:** 2023-01-21

**Authors:** Jawahar Singh, Vishal Varshney, Vishnu Mishra

**Affiliations:** 1 Laboratorio de Genomica Funcional de Leguminosas, Facultad de Estudios Superiores Iztacala, Universidad Nacional Autonoma de Mexico, 54090 Tlalnepantla, Mexico; 2 Govt. Shaheed GendSingh College, Charama, Chhattisgarh India; 3 Department of Plant and Soil Sciences, Delaware Biotechnology Institute, University of Delaware, Newark, DE 19713 USA

**Keywords:** Legumes, AUR1, Cell division, Infection thread, MAP Kinase

## Abstract

We highlight the newly emerged regulatory role of a mitotic kinase AUR1, its activator, and its microtubule-associated proteins (MAPs) in infection thread formation for root nodule symbiosis.

## Introduction

The roots of the Leguminosae family members form an endosymbiotic relationship with rhizobia, a group of nitrogen-fixing soil bacteria, consequently, leads to the formation of nodules as lateral root organs (Roy et al. 2020). Through root nodule symbiosis, legumes not only acquire their nitrogen requirements but also nourish the soil with fixed nitrogen. Legumes fix 60 million tons of nitrogen every year worldwide (Smil 1999), making root nodule symbiosis an environment-friendly alternative to reduce our agricultural dependence on synthetic fertilizers.

Rhizobia in these nodules convert gaseous dinitrogen into forms like ammonium and/or nitrates; that the plant host can absorb in exchange for carbon-rich organic compounds produced by the plant (Roy et al. 2020). The core of this legume-rhizobia symbiosis depends on mutual communication between the legume and rhizobia, which is triggered by flavonoid compounds, released into the soil by legume roots (Oldroyd et al. 2011). This attracts compatible rhizobia and causes them to produce and secrete nodulation factors (NF), which are highly specific lipo-Chito-oligosaccharide signaling molecules (Oldroyd et al. 2011). Legume roots contain LysM receptor-like kinase that helps them to recognize NF signals (Singh and Verma 2022). Two synchronized processes are triggered by NF recognition; (i) the bacterial infection in the epidermis, which allows bacteria to invade host cells, and (ii) the induction of the pericycle and cortical cell division, leading to the formation of the nodule primordia (Murray et al. 2011). The bacteria are enclosed into host membrane-derived structures called symbiosomes as they pass through the infection threads (ITs) into the nodule primordial cells, where they fix nitrogen (Oldroyd et al. 2011). Infection threads are unique cell wall invaginations of plant origin that utilize cell cycle and polar growth processes and serve as a channel for the colonization of bacterial cells. The pre-infection thread, a subcellular structure that arises before infection thread development, was discovered to mimic the phragmosome, an essential step in cell division (Murata and Wada 1997). In each case, a substantial transcellular cytoplasmic bridge develops that predicts the direction of cell wall deposition. The formation of nodules has then evolved through the recruitment of cell divisions and the gradual appearance of new cell types.

The cell cycle is a highly ordered series of events that results in cellular fission and the formation of two daughter cells. The formation of the cell division plane, which involves dramatic changes in the organization of the cytoskeleton, is a critical event for cell division. Interactions with microtubule-associated proteins enable these roles, which include positional regulation of cytoskeletal rearrangements and cytokinesis. Because of the significant role of microtubules in rhizobial infection, including root hair curling to capture rhizobia and infection thread formation, a similarity has always been drawn between these two processes of infection thread formation and cell cycle (Perrine-Walker et al. 2014). In eukaryotes, Aurora kinases (AUR) are identified as mitotic serine/threonine kinases that AURs play multifaceted roles in cell cycle processes, and their importance in mitosis is conserved across eukaryotes. AUR modulates cell division plane formation, cytokinesis, centromere formation, and chromosome segregation (Gutierrez 2016). In addition to mitotic processes, AUR has been shown to regulate protein dynamics in the interphase of animal cells (Weimer et al. 2016). The studies in eukaryotes have demonstrated the interaction of AUR1 with the Xenopus kinesin-like protein 2 (TPX2), which is responsible for phosphorylation and activation of AUR1 (Bayliss et al. 2003; Eyers et al. 2003). Due to their function in cytokinesis and participation in human cancer, AURs have been widely investigated in mammals and yeast; however, there are relatively fewer studies of them existing in the plant system (Goldenson and Crispino 2015). Plant AURs are a small family of genes made up of two groups: (i) α-AURs function in early mitotic processes and the development of bipolar spindles, while (ii) β-AURs appear to be involved in the segregation of chromosomes during anaphase. (Weimer et al. 2016). Both *Arabidopsis* and *Medicago* contains three AURs; however, *Arabidopsis* contains two AURs in α-AURs and one in the β-AURs group, by contrast, *Medicago* contains only one member in α-AURs and two in the β-AURs group. In *Arabidopsis*, *Ataur1aur2* knockdown mutants have been shown to exhibit a defective cytokinesis phenotype that results into randomly oriented cell plates (van Damme et al. 2011). Additionally, activation of the microtubule-associated protein, AtMAP65-1 phosphorylated by AtAUR1 is required for efficient cell cycle progression (Boruc et al. 2017). Previously, it has been shown that AUR1 is involved in legume-rhizobia symbiosis based on its elevated expression levels in legumes during symbiotic relationships with nitrogen-fixing soil bacteria (Breakspear et al. 2014). The role played by AUR and its other interacting partners such as MAPs and TPX2 in the regulation of nodule symbiosis remain elusive.

**Fig. 1:**
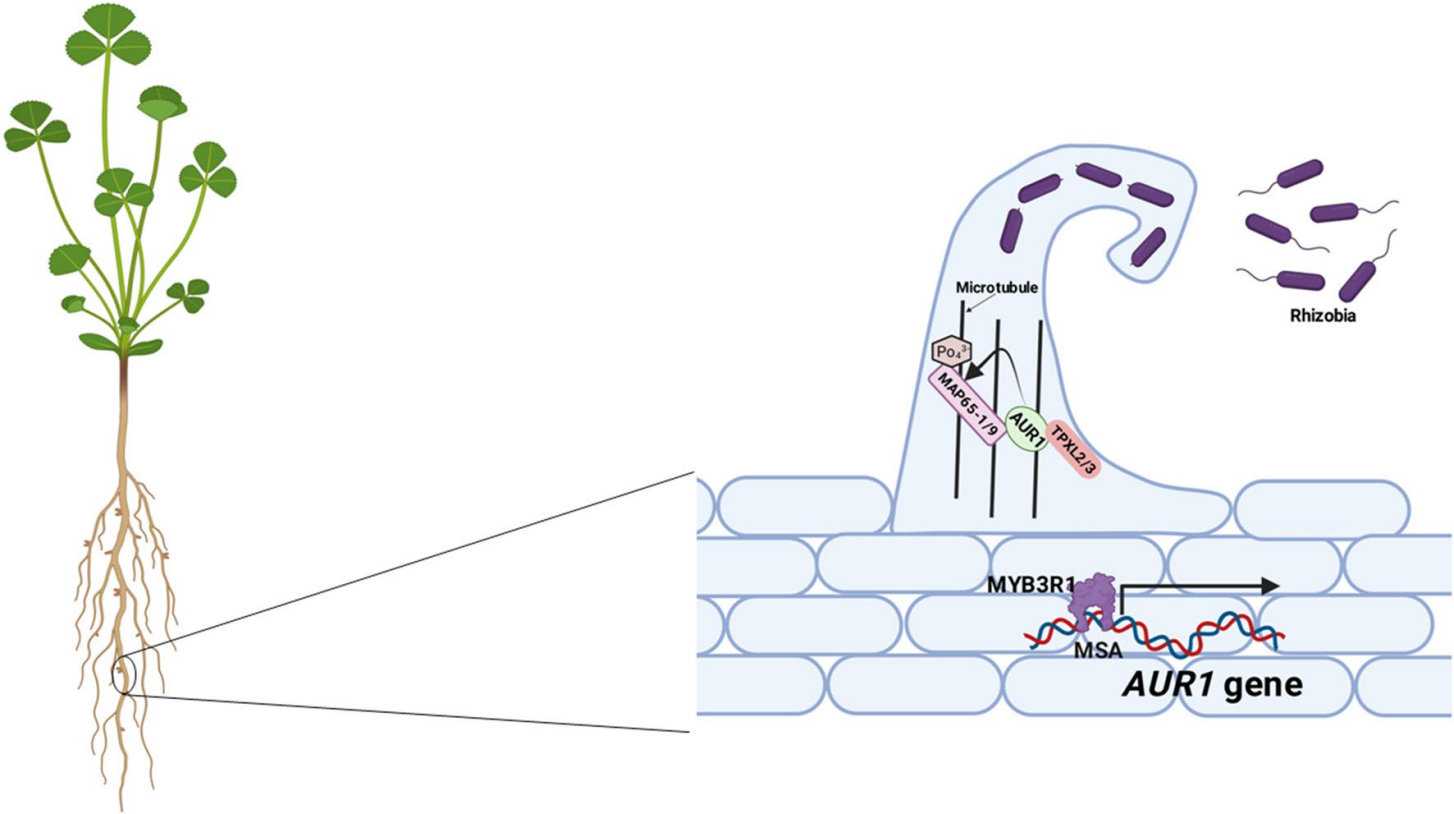
Conceptual model of AUR1-TPX-MAP645 module controlling infection thread formation for legume nodulation. AUR1 interacts with MAP65-1/9, which leads to the phosphorylation of MAP65-1/9 via AUR1 in the presence of TPXL2/3. MYB3R1 binds over the MSA (mitosis-specific activator) elements present over the promoter of *AUR1* for induction of AUR1 for infection thread formation

Interestingly, a recent study by Gao et al. (2022) discovered the role of cell cycle machinery in nodule formation, which promotes transcellular deposition of cell wall material, a feature common to both infection thread formation and cell division. There are three key points of this study (i) AUR1 is involved in microcolony establishment and infection thread progression in root nodule symbiosis; (ii) the TPXL-AUR1-MAP65 module regulates microtubule functions for the formation of infection thread; and (iii) Myeloblastosis (MYB) family protein, MYB3R1 acts upstream of the TPXL-AUR1-MAP65 module to promote rhizobial infection and activate expression of AUR1 (Fig. 1).

## The role of AUR1-TPX-MAP645 in controlling rhizobia infection thread progression

Gao et al., (2022) carried out an expression analysis of genes involved in cell cycle machinery in infected root hairs of *Medicago truncatula* seedlings to examine the role of cell cycle components in root nodule symbiosis. Gene expression analysis revealed that AUR1, cyclin, and cyclin-dependent kinases expressed more in the root hairs of *M. truncatula* seedlings, notably in the infection zone of mature root nodules, lateral root primordia, and root tips. To confirm the role of AUR1 in the early stages of infection, the authors used a novel CRISPR/Cas9-based tissue-specific knockout (KO) method, in which Cas9 expression is controlled by the chalcone-O-methyltransferase 3 (*ChOMT3*) promoter that is exclusively expressed in rhizobia-infected cells. In KO lines, followed by *sinorhizobium* *meliloti* (rhizobial partner of *M. truncatula*) infection the majority of infection threads formed showed abnormal morphologies like multiple branching, merging into one another and some formed balloon-like structures. Also, the extent of rhizobial infection was significantly reduced after *S. meliloti* inoculation in the transgenic *aur1* plant compared to the control. To further support this hypothesis, the authors generated AUR1-DN (a dominant negative version of AUR1) by introducing a mutation in the ATP binding site (Lys66 to Arg) under the *ChOMT3* promoter (*ChOMT3pro: AUR1 -DN*). The transgenic hairy root expressing *ChOMT3pro: AUR1–DN* had higher transcript expression of AUR1 but the resulting AUR1 protein was kinase dead. At 7 day post inoculation (dpi), AUR1-DN lines also showed a significant decrease in infection threads, similar to *aur1* roots. These findings suggest that AUR1 is involved in the formation of infection threads in the early stage of infection.

To elucidate the molecular mechanism of AUR1 involvement in the modulation of infection thread progression during root nodule symbiosis, the authors searched the allies of *AUR1* involved in root nodule symbiosis. In animal systems, the interaction of AUR1 with proteins of the TPX2 family is necessary for its activation during cell division (Tomaštíková et al. 2020; Smertenko et al. 2021). Based on their AUR binding domains, the *M. truncatula* genome was found to contain seven copies of the TPX2/TPXL protein (Gao et al. 2022). Two TPXLs, namely TPXL2 and TPXL3, were found to be symbiotically induced in root hairs and expressed in nodules. The interaction between TPXL2 and TPXL3 and AUR1 was examined and confirmed using the yeast-two-hybrid (Y2H) assay, in vivo co-immunoprecipitation (co-IP), and split luciferase complementation assays (Gao et al. 2022). In addition, TPXL2 and TPXL3 share the same localization pattern as AUR1 i.e., in the endoplasmic reticulum (ER) and nuclei of root hairs of rhizobia-inoculated seedlings (Gao et al. 2022).

AtTPXL3-activated *AtAUR1* was found to regulate microtubule dynamics during cell division in *Arabidopsis* by phosphorylation of the microtubule-associated protein AtMAP65-1 (Boruc et al. 2017). In eukaryotes, the MAP65 protein family functions in tubulin cross-linking and cytokinesis (Boruc et al. 2017). According to Gao et al. (2022), rhizobia and NFs stimulate the expression of MAP65-1 (Medtr5g093860) and MAP65-9 (Medtr6g061690) in root hairs. Furthermore, the interaction and subcellular localization studies confirmed the interaction between AUR1 and MAP65-1/9, which leads to the phosphorylation of MAP65-1 via AUR1 in the presence of TPXL2 or TPXL3. These results confirmed the involvement of conserved TPXL-AUR1-MAP65 module in rhizobia infection.

To bring more functional significance to this module, the regulation of AUR1 was explored for root nodule symbiosis. The majority of rhizobia-induced genes require the master regulator *Nodule Inception* (*NIN*) gene, but *AUR1* is an exception, as its expression in *nin* mutants is more than double that in wild-type. In *M. truncatula* and 20 other legumes species including *Lotus japonicus*, *Phaseolus vulgaris*, *Glycine max, Pisum sativum, Cicer arietinum*, etc., examination of *AUR1* promoter sequence revealed the presence of adjacent mitosis-specific activator (MSA) *cis*-elements (AACGG) to the transcription start sites. MSA elements are transcription factor binding sites for the MYB3R (R1R2R3-Myb) transcription factor family, which is found in all eukaryotes and plays an important role in cell division (Haga et al. 2011). MYB3R1 was found to act as a transcription activator that binds to the *AUR1* promoter region for its activation. To investigate the functional role of MYB3R1 in nodule formation, a construct containing MYB3R1 linked to a plant-specific, ERF-associated amphiphilic repression domain (SRDX) that transforms transcriptional activators into potent repressors was used to generate the hairy roots of *M. truncatula.* Loss of MYB3R1-SRDX in hair roots decreased AUR1 expression and generated fewer infection threads. However, the number of infection threads increases when MYB3R1 is overexpressed. These results suggest that MYB3R1 is involved in the development of infection thread by acting upstream of the TPXL-AUR1-MAP65 nodule to promote rhizobial infection.

### Concluding remarks

In short, this study comprehensively uncovered the role of AUR1 in the model legume *Medicago*. AUR1 and MAPs are required for intracellular infection. Authors found TPXL and MAP65 as AUR1 interaction partners and MYB3R1 as an AUR1 transcription regulator. This advancement is a critical step in understanding the cell cycle components involved in endosymbiotic infection in root nodule symbiosis and in further exploring and characterizing the other components of the cell cycle in root nodule symbiosis.

### Future direction

The increased expansion of our knowledge about the molecular and cell biology of legume nodules has also raised a number of new questions. First, more studies need to be done to better understand the conserved role of the cell division machinery in nodule formation like how the different cell cycle components have evolved in two different types of nodules namely determinant and indeterminate nodules. Secondly, the study of AURs in plants has been largely limited to the model legume such as *M. trancatula* only, so exploring the role of AUR1 and its associated mechanism in economically important crops like *Cicer aeritinum*, and *Phaseolus vulgaris* would be very exciting. Thirdly, it is well known that components of the exocyst complex play an important role in infection thread formation (Liu et al. 2019), so it will be interesting to see if there is any interplay among the components of the exocyst complex and the cell cycle components, and if yes then how they regulate infection thread and nodule symbiosis together. Finally, several mitotic genes expressed during infection remain to be studied and should be explored to elucidate how and why this deeply conserved and fundamental program was co-opted into symbiosis.

## Data Availability

The authors declare that no new data were generated in this article.
